# Antecedents for Older Adults’ Intention to Use Smart Health Wearable Devices-Technology Anxiety as a Moderator

**DOI:** 10.3390/bs12040114

**Published:** 2022-04-18

**Authors:** Mei-Yuan Jeng, Fan-Yun Pai, Tsu-Ming Yeh

**Affiliations:** 1 Department of Life Sciences, National Open University, New Taipei City 247, Taiwan; 980020@gapps.nou.edu.tw; 2 Department of Business Administration, National Changhua University of Education, Changhua 500, Taiwan; 3 Department of Industrial Engineering and Management, National Quemoy University, Kinmen 892, Taiwan

**Keywords:** technology readiness, technology interactivity, technology anxiety, smart health wearable device

## Abstract

The increase in the demands for surveillance of chronic diseases, long-term care, and self-health management has allowed mobile smart health wearable devices to become products with greater business potential in past years. Wearable devices being able to be worn for long periods are the most suitable for 24-h weatherproof monitoring. Nevertheless, most technological products are not developed specifically for older adults. Older adults might be apprehensive and fearful about the use of technological equipment and might appear “technologically anxious”, so it was wondered whether older adults could smoothly operate and comfortably use smart wearable device products, and how “technological anxiety” would affect their behavior and attitude towards using these devices. The variables of “technology readiness”, “technological interactivity”, “perceived usefulness”, “perceived ease of use”, “attitude”, and “intention to use” are therefore discussed in this study. Taking “technological anxiety” as the moderating variable to develop the questionnaire scale, the quantitative research with structural equation model is applied to discuss the older adults’ intention to use smart health wearable devices. The questionnaire was distributed to older adults’ community care centers, senior centers, and senior learning centers in Taiwan, and to an older adults’ group above the age of 60 with experience in using smart bracelets. A total of 200 questionnaires were distributed, and 183 were retrieved, with 166 valid copies. The research results reveal that users with higher technology readiness, and older adult users with higher technological interactivity, present a higher perceived ease of use and perceived usefulness. Technological anxiety would affect users’ attitude and further influence the intention to use. The research results could help understand older adults’ needs for using smart health wearable devices.

## 1. Introduction

Along with the boom and diversification of technological products, new man–machine digital product technology provides different communication, entertainment, and care and is able to interact with the older adults group [1]. Mobile health devices (e.g., smart phones, smart watches) use mobile technology, wireless equipment, and sensors [2,3]. By wearing devices during daily activities, the accurate physiological, psychological, and emotional information as well as environmental status could be acquired through the sensors outputting data and the added self-reported data [4,5]. The behavioral surveillance with individualized feedback could track users’ health conditions, including sleep and psychological/physiological data of calories burned, heart rate, brain activity, and muscle action [6,7]. Such devices used for health-related surveillance present higher precision than traditional sampling frequency and are more suitable for long-period digital preventive measure and self-evaluation of behavior [8,9]. Since Apple promoted Apple Watch, the media started to pay attention to smart wearable devices. In face of the problems of low birth rate and aging society, smart wearable devices with medical technology would play the critical roles of care and accompaniment [10].

With the rapid adoption of smart technologies in people’s daily lives, utilizing smart technologies, such as wearables, service robots, Internet of Things (IoT) applications, and other home devices to satisfy the demand and improve the quality of life of older adults has become more necessary [11]. The boom and diversification of technological products, either software or hardware equipment, result in convenience of people’s life [12]. The design of wearable products is also advanced and has promoted the relationship between people and technological products to be closer. The design of technological products, including battery life, interface display design, man–machine interactivity, and diversified software, enhances people’s quality of life [13]. For older adults, such technological products would result in changes in life, products, or environment. A lot of research therefore has started to discuss the issue related to older adults in an environment with technological products, e.g., a study on older adults’ smart home [14]. Such research provides the smart living environment with digital health surveillance, safety protection, and medical interaction for older adults [8,15]. It reveals the importance and develops the ability of research on older adults and man–machine technological products.

Wearable technology consists of clothing and accessories that incorporate advanced technologies to assist individuals wearing them to perform their daily tasks quickly and efficiently [3]. Such technologies are very fundamental to monitoring the physiological data of older people or individuals with chronic conditions and facilitating timely clinical interventions [1]. Past research also proved the application of smart wearable devices to disease management and prevention, such as diabetes control [16], treatment of depressive disorder [17], hypertension control [18], and assessing function in Alzheimer’s disease [19]. Such preventive measures were verified in clinical research. Burke et al. [20] preceded diet control with PDAs to reduce calorie intake. Schoeppe et al. [21] proved that the use of smart phone applications and a training platform could effectively improve diet as well as physical activity and sedentary prevention behaviors. Kekade et al. [5] surveyed 233 seniors and discovered that more than 60% of older adults were interested in the use of smart wearable devices and expected to use them for improving physical and psychological activities. Tison et al. [22] used a commercially available smartwatch to detect atrial fibrillation. Ray et al. [23] revealed that GPS-based wireless tracking devices, such as Fitbit, Mi band, Oura ring, and Alice PDx, have been preferred and widely used by people with dementias. Ogundaini et al. [24] explored opportunities for integrating mobile health Information and Communication Technologies (ICTs) into the clinical settings of hospitals in South Africa and Nigeria.

Smart wearables are equipped with sensors and transmitters to monitor, collect, display, and transmit data automatically and perpetually [25]. Nevertheless, there are challenges to apply smart wearable technology to smart healthcare, including standard certification, continuous usability, users’ intention to use [26], shortage of cost-effective wearable sensors, heterogeneity of wearable devices connected, and high demand for interoperability, which are the bottlenecks encountered for smart wearable devices in the healthcare market [27]. Most technological products are not developed specifically for older adults [28]. Moschis [29] indicated that elder consumers presented different needs and preferences from the young generation and elder consumers would be less affected by the media and information technology. Adults aged above 50 would pay more attention to convenience, choose products with brands, and be willing to pay more for better product quality or service. Elder consumers seemed to dislike risks but prefer products and service with better safety [30]. Kruse et al. [31] pointed out independence, comprehension, and visibility as the factors in older adults using technological products, while complexity and limited availability as the obstructive factors. Smart wearable devices are helpful for older adults’ health control [1,2,25], but the products have to show obvious advantages for older adults to accept mobile health devices and must conform to the older adults’ goals, expectations, and lifestyles [32]. Shieh et al. [33] studied wearable equipment for monitoring sleep, with a questionnaire survey, to discuss older adults’ opinions about wearable equipment. The results revealed that older adults with distinct backgrounds showed different opinions about wearable equipment.

From previous literature, “technology readiness”, “technology interactivity”, “perceived usefulness”, “perceived ease of use”, “attitude”, and “intention to use” as well as the moderating variable of “technology anxiety” are used in the proposed conceptual model as the antecedents of intention to use health wearable devices for older adults. A questionnaire scale of each variable was developed to analyze older adults’ intentions to use smart health wearable devices. The research results could help the understanding of older adults’ needs for the use of smart health wearable devices as well as understand the physical and mental health management and successful aging of older adults.

## 2. Literature Review

### 2.1. Older Adults’ Usage Intention and Technology Anxiety

Healthcare, being an important issue in aging society, is a key factor in older adults’ quality of life [1]. It would have older adults consider obvious advantages of products and would meet the goals, expectations, and lifestyles to have them accept smart health wearable devices [32]. International Society for Gerontechnology (ISG) covers health, housing, mobility, communication, leisure, and work in “gerontechnlogy” [34]. From the viewpoint of technology, it is important to help older adults with declined physical functions to live with health, comfort, safety, and dignity through technologies [35,36]. The most important challenge for industries is not continuously developing new products but trying to have older adults using smart technological products in daily life [27,37]. Kruse et al. [31] discovered in their research that independence, comprehension, and visibility were the factors in enhancing older adults using technological products, while complexity and limited availability were the obstructive factors. However, most technological products were not developed specifically for older adults, and the technology operation required learning and skills. Holzinger et al. [38] compared the use of smart phones between youth and older adults and concluded a high heterogeneity of learning time, performance speed, error rate, and subjective satisfaction between older adults and youth. As a result, in consideration of possible physiological and psychological limits, technological products designed for older adults should be simple and easy to operate [28].

Davis [39] considered that users’ acceptance of information or technology was affected by perceived usefulness and perceived ease of use. A technology acceptance model (TAM) was then constructed to explain and predict the user model under the acceptance of information system. TAM initially discussed interviewees’ acceptance of the use of information system, from users’ cognition. The successive researchers applied the model or the modified model to discuss the acceptance of electronic and digital products or other information systems [2,5,10,13,33,35,40].

Parasuraman [41] proposed the technology readiness index (TRI) to discuss the relevance between personality traits and intention to use technological products, i.e., individual willingness to accept new technologies and the intention to use for work or life, particularly for understanding consumers’ readiness for the use of computers and network. Technology readiness was divided into the four dimensions of optimism, innovativeness, discomfort, and insecurity to represent individual readiness for using technologies. Both optimism and innovativeness were the driving force to use innovative technological products and could be regarded as the positive perception of using technology; discomfort and insecurity, on the other hand, referred to the resistance to the idea of using innovative technological products, as the negative perception of using technologies. Positive driving force and negative resistance might simultaneously exist in an individual and affect users’ technology acceptance and use behavior [42].

Chang et al. [43] considered that factors in users deciding to use smart wearable devices were not defined and were still at the early stage in the market development. They applied TAM to discuss important factors in 342 participants’ smart wearable device use behavior and discovered that users did not expect smart wearable devices to provide telecommunication functions or become fashion items. With wearable equipment for monitoring sleep as the target product, Shieh et al. [33] applied a questionnaire survey to discuss older adults’ opinions about the form of smart wearable devices. The results revealed that older adults with different backgrounds presented distinct opinions about the form of wearable equipment. Taking 233 elders as examples, Kekade et al. [5] discovered that more than 60% of elders were interested in using smart wearable devices in the future and expected to use them for improving physical and psychological activities.

Tsai et al. [44] regarded technology anxiety as apprehension and fear of using technological equipment, i.e., whether older adults could accept and smoothly operate and comfortably use smart wearable device products against negative psychological perception of nervousness, fear, and self-doubt when learning new skills. When older adults find obstacles in using new technological products, the effect of “technology anxiety” on the use behavior and attitude is the research motivation in this study.

### 2.2. Derivation of Hypotheses

Technology would affect users’ intention to use and evaluation, and user factors would also influence the willingness to adopt and accept new technology. Ajzen and Fishbein [45] mentioned possible external variables to affect users’ behavioral intention, including demographic characteristics, attitudes towards a specific target, and personality traits, where “personality traits” were user factors. The idea was similar to TRI in that users’ personality traits would affect the willingness to accept new technology. Ahmad et al. [46] indicated that individual technology readiness, when using a new technology, contained the basic concept of the new technology and the ambition to use new technology; the higher technology readiness revealed the strong interests in and ability of new technology such that the intention to use would be enhanced. Under strong technology readiness, the concept about new technology would be better. Past research proved the significant effect of technology readiness on perceived usefulness. Chen et al. [26], Ahmad et al. [46], and Widyawan and Santosa [47], with TRI as the external variable of TAM, found out remarkable relations between TRI and perceived usefulness and perceived ease of use in TAM. TRI is also used as the external variable of TAM in this study to discuss the older adults’ intention to use wearable devices. The hypotheses are therefore proposed in this study.

**Hypothesis** **1** **(H1).** *Technology readiness shows positive effects on perceived ease of use.*

**Hypothesis** **2** **(H2).** *Technology readiness reveals positive effects on perceived usefulness.*

Older adults being able to use various functions of smart health wearable devices as desired stands for older adults realizing that they do not need to pay extra efforts for using the interactive function of devices, could rapidly and easily use various interactive mechanisms, and simply exchange information with others through such interactive mechanism to increase knowledge and solve problems. Interactivity is the most important characteristic in a smart health wearable device system and shows positive effects on perceived ease of use [48]. Webster and Ho [49] pointed out interactivity as interaction ability between people and systems or feedback acquired from systems. Hsu et al. [50] considered that interactivity contained real-time and helpful information. Smart health wearable devices could provide information like number of steps, calories consumed, running route, heart rate, and blood pressure, which could be easily acquired by older adults, as useful information for self-health management [11]. Accordingly, hypotheses are proposed in this study.

**Hypothesis** **3** **(H3).** *Technology interactivity shows positive effects on perceived ease of use.*

**Hypothesis** **4** **(H4).** *Technology interactivity presents positive effects on perceived usefulness.*

Davis [39] proposed a technology acceptance model and pointed out positive effects of perceived ease of use on perceived usefulness. In the technology acceptance model research review, Hung et al. [51] organized and classified journals using the technology acceptance model and discovered that 30 pieces of literature, among 39, showed positive effects of perceived ease of use on perceived usefulness. Hypothesis is then proposed in this study.

**Hypothesis** **5** **(H5).** *Perceived ease of use shows positive effects on perceived usefulness.*

TAM of Davis et al. [39] and Islam et al. [52] considered that website nature (e.g., perceived usefulness and perceived ease of use) would affect online users’ participation attitude. Pai and Yeh [48] mentioned that individuals with higher perceived usefulness of specific information technology would present positive attitude towards technology. Consequently, hypotheses are proposed in this study.

**Hypothesis** **6** **(H6).** *Perceived ease of use reveals positive effects on attitude.*

**Hypothesis** **7** **(H7).** *Perceived usefulness shows positive effects on attitude.*

In the explanation of online consumer behavior, Pavlou and Fygenson [53] revealed the effect of attitude on intention to use. Bruner II and Kumar [54] discovered that users with positive perception of information technology or achieving the engaged behaviors through information technology would show higher possibility to use information technology. Older adults being aware of using smart health wearable devices for self-health management would affect the intention to use. As a result, another hypothesis is proposed in this study.

**Hypothesis** **8** **(H8).** *Attitude presents positive effects on intention to use.*

From the viewpoint of technology, it is important to help physical-function-declined older adults live with health, comfort, safety, and dignity through technology; however, older adults might discover some obstacles when facing the use of technological products [12,35]. Researchers have found that technological anxiety and resistance to change impact geriatric technology uptake [1]. Technology anxiety refers to apprehension and fear of using technological equipment [44]. Lin [55] indicated that individual emotional reactions of fear and nervousness during the use of or planning to use computers would affect the attitude towards computer products. Jeng et al. [56] defined computer anxiety as individual uncomfortable, scared, nervous, or worrying reactions when learning how to use computers, using computers, or expecting to come into contact with computers. Such reactions might be the negative attitudes towards computers accompanied with physiological and psychological discomfort to further hinder the computer learning or use in the future. As a consequence, the following hypothesis is proposed in this study.

**Hypothesis** **9** **(H9).** *Technology anxiety shows moderating effects on attitude and actual intention to use.*

## 3. Research Method

### 3.1. Research Structure

**Figure 1:**
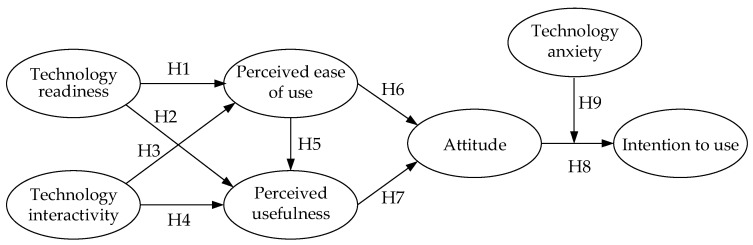
Concept model of the older adults’ behavioral intention to use smart health wearable devices.

In consideration of the older adults being able to accept, smoothly operate, and comfortably use smart health wearable device products, the effect of “technology anxiety” on use behavior and attitude is studied, when they cannot use such new technological products. Using variables of “technology readiness”, “technology interactivity”, “perceived usefulness”, “perceived ease of use”, “attitude”, and “intention to use” and moderating the variable of “technology anxiety” as well as referring to relevant literature, the hypotheses are established for testing. With a literature review, the research structure is proposed as in Figure 1.

### 3.2. Questionnaire Design

With the questionnaire survey, two parts are included. According to domestic and international researchers’ research points of view and referring to relevant literature, the item contents are designed, and the questionnaire contents are analyzed, with 7 scales (Table 1). The technology readiness scale aims to understand users’ optimism, innovativeness, discomfort, and insecurity. The technology interactivity scale aims to understand older adults’ use of interactive communication with health devices, including feedback, control, entertainment, and connection. The perceived usefulness scale aims to measure user perception of information provided by smart health wearable devices enhancing living convenience. The perceived ease of use scale aims to understand the degree of users considering smart health wearable devices being easy to operate. The attitude scale is used for measuring users’ perception and evaluation of smart health wearable devices. The intention to use scale measures users’ intention to use health devices. The technology anxiety scale measures older adults’ apprehension and fear about using technological equipment, e.g., anxiety about equipment operation and information exposure. Detailed questionnaire items are shown in Appendix A Table A1.

The second part shows demographic variables, covering users’ gender, education, age, marriage, occupation, living situation, average monthly disposable income, experience in using smart phones/tablet for recording or measuring health conditions, experience in using smart health wearable devices, and willingness to use smart health wearable devices. With Likert’s 5-point scale, five options of “extremely agree”, “agree”, “ordinary”, “disagree”, and “extremely disagree” are scored 5, 4, 3, 2, and 1. After the preliminary design of the questionnaire, we sent the questionnaire and procedure to the Research Ethics Committee of National Cheng Kung University, Taiwan, for ethical review. This study was conducted under approval number No.108–184.

### 3.3. Questionnaire Sample

The questionnaire was distributed to older adults’ community care centers, senior centers, and senior learning centers in Taiwan, and to an older adults’ group above the age of 60 with experience in using smart bracelets. We invited older adults to provide their opinions about smart bracelets first. If the older adults agreed, we provided them smart bracelets. After using the devices and providing opinions, the recruited samples could keep the devices as gifts.

In consideration of older adults’ eyesight and responding ability, the interviewer read out the questions one-to-one to them, who then answered with agree, disagree, extremely disagree, ordinary, or extremely agree, according to their experience. The interviewer then ticked the answer. Some older adults became impatient in the process and quit. A total of 200 copies of questionnaire were distributed, and 183 copies were retrieved, with 166 valid copies.

## 4. Research Result

### 4.1. Description of Demographic Variables

The effective questionnaire samples contain responses from 74 males (44.6%) and 92 females (54.4%), where the age group of 60–69 appears the most at 119 (71.6%), followed by 39 people aged 70–79 (23.5%). Eighty people show having the education of university or above (48.2%), followed by 44 with senior high (vocational) school (26.5%). Fifty-seven people are in the service industry (34.3%), followed by 50 people who are retired (30.1%). Seventy people show their monthly disposable income to be 20,000–40,000 NT dollars (42.2%), followed by 44 with 40,001–60,000 NT dollars (26.5%). Up to 102 people live with family members (61.4%), followed by 55 living with a spouse (33.1%). Ninety-nine people have experience in using smart health wearable devices (59.6%). An amount of 123 people show the volunteer willingness to use smart health wearable devices (74.1%). Detailed information is shown in Table 2.

### 4.2. Reliability Analysis

Cuieford [64] proposed that the Cronbach’s α reaching above 0.7 was high reliability, between 0.35–0.7 as medium reliability, and lower than 0.35 as low reliability. Nunnally [65] suggested that the Cronbach’s α reaching above 0.7 was acceptable. The Cronbach’s α of dimensions shows technology readiness at 0.76, technology interactivity at 0.91, perceived usefulness at 0.82, perceived ease of use at 0.81, attitude at 0.81, intention to use at 0.86, and technology anxiety at 0.87, higher than 0.7. It reveals certain reliability of the questionnaire (Table 3).

Along with an approaching aging society, information technology users are expanded to older adults’ groups who do not frequently use networks to manage their health problems through smart health wearable devices. Aiming at the relations among technology readiness, technology interactivity, perceived usefulness, perceived ease of use, attitude, and intention to use of older adults’ use of smart health wearable devices, the moderating effect of technology anxiety on attitude and intention to use are discussed in this study. In terms of seven variables for this study, older adults show the overall evaluation of 3.932 for technology readiness for a smart health wearable device, revealing that older adults adapt to and are optimistic to use new technologies for mastering their health status, and smart bracelets do not need much time for learning or are not difficult to use. Older adults present the overall evaluation of 4.238 for technology interactivity of smart health wearable devices, explaining that older adults regard the use of smart health wearable devices and being able to rapidly inquire about the desired information is interesting and pleasant. Users show the overall evaluation of 4.289 for perceived usefulness of wearable devices, showing that older adults regard the use of smart health wearable devices as being able to increase the efficiency in health control and effectively improve health to promote the quality of life. The overall evaluation of 3.767 for perceived ease of use reveals that the smart health wearable device interface is easy to operate, and the multiple functions catch older adults’ eyes; however, unsuitable interface design for older adults would be refused, the same results as Choi and DiNitto [66].

The overall evaluation of 4.018 for attitude shows the positive evaluation of users after the use of wearable devices. When older adults consider that the use of technology can acquire useful information of a healthy life and provide entertainment, they will not easily refuse to use new technological information. The overall evaluation of 4.285 for intention to use reveals older adult users’ positive willingness to continuously use smart health wearable devices in the future. Moreover, the overall evaluation of 3.708 for technology anxiety shows that older adult users of smart health wearable devices worry about breaking smart bracelets in the use process and not being able to properly protect personal health information. The safety of technology use is related to personal learning anxiety (e.g., dealing with operation systems, solving problems, and worrying about destroying expensive equipment) [67]. Older adults would reduce the sources of absorbing technological information after retirement and are afraid of coming into contact with information products. In this case, more efforts should be made for the design of smart bracelets/watches and wearable devices; particularly, older adult users’ needs should be emphasized to provide diverse functions and multiple applications to have smart bracelets and watches become smart health wearable devices with high popularity and to integrate into each older adult user’s life, as a part of their life.

### 4.3. Correlation Analysis

The internal variables of technology readiness, technology interactivity, perceived usefulness, perceived ease of use, and attitude show their significance (*p* < 0.05). Although there is not a limit to high correlation, a coefficient higher than 0.90 should be particularly paid attention to. Correlation coefficients being higher than 0.80 might demonstrate multicollinearity. The correlations among the seven variables in this study appear in −0.90–0.751, without collinearity, and all variables achieve the significance (*p* < 0.01), revealing good correlations [68], Table 4.

### 4.4. Test of Research Hypothesis

There are nine hypotheses in this study. Multiple linear and hierarchical linear regression were used to test the proposed hypotheses. Multiple linear regression and path analysis were used to test H1 to H7 (Table 5). Otherwise, hierarchical linear regression was employed to test H8 and H9 (Table 6).

The research results are concluded as followings. H1: Technology readiness showing positive effects on perceived ease of use is supported (*β* = 0.385; *t* = 5.344; *p* < 0.001). It reveals that users with higher new technology readiness would feel it easier to learn and use technology. H2: Technology readiness presenting positive effects on perceived usefulness is supported (*β* = 0.545; *t* = 8.316; *p* < 0.001). It proves that users with higher new technology readiness can feel the enhancement of quality of life and health through technology. H3: Technology interactivity appearing positive effects on perceived ease of use is also supported (*β* = 0.511; *t* = 7.620; *p* < 0.001), revealing that users with higher new technology interactivity could more rapidly inquire for the desired information through smart health wearable devices.

H4: Technology interactivity presenting positive effects on perceived usefulness is supported (*β* = 0.751; *t* = 14.552; *p* < 0.001), revealing that users with higher new technology interactivity would be interested in the use of smart health wearable devices. H5: Perceived ease of use showing positive effects on perceived usefulness is supported (*β* = 0.461; *t* = 6.658; *p* < 0.001), revealing that users perceiving smart health wearable devices being easy to use would present higher use effectiveness. H6: Perceived ease of use showing positive effects on attitude is also supported (*β* = 0.579; *t* = 9.104; *p* < 0.001), showing that users perceiving smart health wearable devices being easy to use would present higher intention to use.

H7: Perceived usefulness shows positive effects on attitude. The empirical research results prove significantly positive effects of perceived usefulness on attitude (*β* = 0.600; *t* = 9.611; *p* < 0.001), revealing that users regarding the use of wearable devices being able to enhance the performance would demonstrate a higher intention to use.

As for H8 and H9, hierarchical regression was used for hypotheses testing. Hierarchical regression is a way to show if added-in variables can explain a statistically significant amount of variance in dependent variables after accounting for all other variables. It is usually used to test the effects of moderation variables. The hierarchical regression analysis results are presented in Table 5.

H8: Attitude presents positive effects on intention to use. The remarkably positive effects of attitude on behavioral intention are proven in this study (*β* = 0.647; *t* = 10.866; *p* < 0.001), showing that users with higher intention to use would have higher willingness to use. H9 verifies the moderating effect of technology anxiety on attitude and intention to use, with technology anxiety as the moderating variable. The effect of the interaction of attitude and technology anxiety on intention to use is analyzed with hierarchical regression analysis.

Model 1 shows notable correlations between attitude (*β* = 0.647, *p* < 0.001) and intention to use. Model 2 adds the moderating variable of technology anxiety which could directly and significantly affect intention to use (*β* = 0.221, *p* < 0.001). The interaction between attitude and technology anxiety is further discussed in Model 3. It is discovered that attitude and technology anxiety show remarkable effects on intention to use (*β* = −0.191, *p* < 0.001) (Table 5). In this case, technology anxiety presenting moderating effects on the effect of attitude on intention to use is supported. It reveals that users with stronger anxiety would show negative relations with attitude to further affect the intention to use. The empirical results show that the nine hypotheses in the research model are supported (Table 6).

## 5. Conclusions and Suggestion

Factors in older adults using smart health wearable devices are verified in this study. The first finding is that users with higher technology readiness show higher perceived ease of use and perceived usefulness, i.e., promoting users’ positive attitude to enhance the intention to use. Aiming at users’ technology readiness to segment target groups, optimistic and innovative older adult users could have innovative products promoted to them through various marketing channels. For uncomfortable and insecure users, reinforcing the product demonstration before purchase allows users to rapidly adapt to new products, and the system security of wearable device products should be strengthened. Older adults could easily acquire disease management through mobile software; however, how to define the shared contents or objects would involve personal privacy. Otherwise, the use of technological products would result in negative effects. For example, using video games for exercise might enhance the risk of falls due to limb incoordination or dizziness, worsening eyesight, and fatigue.

The second finding is that older adult users with higher technology interactivity present higher perceived ease of use and perceived usefulness, i.e., promoting users’ positive attitude to further increase the intention to use. For this reason, it is necessary to reinforce the interactivity of smart health wearable devices, perceived ease of use, and perceived usefulness. Technology is strange for most elders such that older adults’ attitudes will be conservative. Nonetheless, after the intervention, the attitude would become positive. When using technologies for acquiring information becomes easy, it reinforces the contact with family members and friends and strengthens the link with society to have older adults be more confident in their self-behavioral ability. The operation interface of wearable devices therefore should be simple and user-friendly, allowing users to perceive the operation or use to be simple. Enterprises and wearable device developers should provide products and services for users’ real needs to enhance users’ dependency on product functions and further affect the intention to use. Accordingly, brand value should be created to reduce users’ technology anxiety. When designing wearable device products, enterprises should reduce the error rate of products. Errors could result in users’ anxiety and negative effects to refuse the use. That is, enterprises and wearable device developers should enhance the interface design and product function of smart bracelets and watches, provide differentiated service, and reinforce marketing and promotion for users’ willingness to continuously use smart bracelets and watches. It should allow consumers finding out product value and reducing technology anxiety to enhance their attitude towards and intention to use wearable devices.

Older adults aged above 60 are the research objects in this study. The research population is people in Taiwan using smart bracelets and watches. Purposive sampling is applied to the survey, which might result in sampling bias. Surveying smart bracelet/watch users such that the research result is inferred to other groups and wearable devices requires further verification. A questionnaire survey is utilized for this study. Being restricted to time and budget, only cross-sectional study data are used for inference and verification. There is not comprehensive research for collecting data to discuss the cause-and-effect relationship among variables. In this case, merely the phenomenon at certain time points is observed, which cannot comprehensively understand the change of users’ attitude and intention to use at different times. The research results are therefore limited to the inference. Successive research is suggested to include qualitative research by an in-depth interview with older adults and different analysis methods to better understand the critical factors in older adult users’ intention to use smart health wearable devices. With the available budget and time, the successive researchers are suggested to survey consumers at different time points and discuss the changes in variables for more effective and reasonable results.

## Figures and Tables

**Table 1:** Operational definitions of variables and sources of reference.

Dimension		Operational Definition	Reference
technology readiness	optimism	Users present positive opinions about technology and believe that technology could enhance the control, flexibility, convenience, and efficiency of daily life.	Parasuraman [41]
	innovativeness	Users’ intention to become the pioneers of technology or thought leaders.	
	discomfort	Users are aware of not being able to control technology and show the feeling of being overwhelmed by technology.	
	insecurity	Showing insecurity about new technology, worrying about confidentiality and privacy, and not trusting the correct operation of technology.	
technology interactivity	feedback	Whether smart health wearable devices could respond to users’ demands.	Cyr et al. [57], Lee [58]
	control	Users could select and control the content and item selection of smart health wearable devices.	
	entertainment	Smart health wearable devices could attract people’s interests.	Dholakia et al. [59]
	connection	Users share experiences in smart health wearable device products or service with others.	Cyr et al. [57], Lee [58]
perceived usefulness		The degree of users perceiving the information provided by smart health wearable devices being able to enhance convenience in life.	Davis [39]
perceived ease of use		The degree of users regarding smart health wearable devices being easy to operate.	
attitude		Users’ perception and evaluation of smart health wearable devices.	Amoako-Gyampah and Salam [60], Ahn et. al. [61]
intention to use		Users’ intention to use smart health wearable devices.	Ahn et. al. [61], Vijayasarathy [62]
technology anxiety	anxiety about equipment operation	Users’ feelings of fear, worry, or expectation when considering to use or using smart health wearable devices.	Spagnolli et al. [6]
	anxiety about information exposure	Users’ fear and worry about private data or information being actively or passively publicized during the use of smart health wearable devices.	Spagnolli et al. [6], Schwaig et al. [63]

**Table 2:** Description of the interview sample.

Category	Item	Number of People	Percentage
Gender	Male	74	44.6%
	Female	92	54.4%
Age	60–69	119	71.6%
	70–79	39	23.5%
	Over 80	8	4.8%
Level of education	Elementary school	10	6%
	Junior high school	32	19.3%
	Senior high school and vocational	44	26.5%
	University or above	80	48.2%
Occupation	Military and government personnel	36	22.7%
	Service industry	57	34.3%
	Manufacturing industry	23	13.9%
	Retirees	50	30.1%
Monthly disposable income	NT $20,000 or less	37	22.3%
	NT $20,001–NT $40,000	70	42.2%
	NT $40,001–NT $60,000	44	26.5%
	More than NT $60,001	15	9.0%
Housing situation	Living with spouse	55	33.1%
	Living with family members	102	61.4%
	Living alone	9	5.5%
The experience in using smart health wearable devices	Yes	99	59.6%
	No	67	40.4%
Willingness to use smart health wearable devices	Volunteer	123	74.1%
	Family request	43	25.9%

**Table 3:** Variable reliability analysis.

Variable	Mean	Standard Division	Cronbach’s α
technology readiness	3.932	0.442	0.76
technology interactivity	4.238	0.514	0.91
technological ease of use	3.767	0.655	0.81
technological usefulness	4.289	0.622	0.82
attitude	4.018	0.629	0.81
intention to use	4.285	0.696	0.86
technology anxiety	3.708	0.697	0.87

**Table 4:** Correlation analysis among variables.

Variable	Technology Readiness	Technology Interactivity	Perceived Usefulness	Perceived Ease of Use	Attitude	Intention to Use	Technology Anxiety
technology readiness	1						
technology interactivity	0.669 **	1					
perceived usefulness	0.545 **	0.751 **	1				
perceived ease of use	0.385 **	0.511 **	0.461 **	1			
attitude	0.506 **	0.693 **	0.600 **	0.579 **	1		
intention to use	0.475 **	0.718 **	0.658 **	0.341 **	0.647 **	1	
technology anxiety	0.531 **	0.299 **	0.262 **	−0.90 *	0.156 *	0.317 **	1

*: *p* < 0.05; **: *p* < 0.01.

**Table 5:** Research hypothesis test result.

Hypothesis	*β*	*t*	*F*	Support
H1: Technology readiness shows positive effects on perceived ease of use	0.385	5.344 ***	38.564 ***	Yes
H2: Technology readiness reveals positive effects on perceived usefulness	0.545	8.316 ***	69.148 ***	Yes
H3: Technology interactivity appears perceived positive effects on ease of use	0.511	7.620 ***	58.072 ***	Yes
H4: Technology interactivity presents positive effects on perceived usefulness	0.751	14.552 ***	211.750 ***	Yes
H5: Perceived ease of use shows positive effects on perceived usefulness	0.461	6.658 ***	44.333 ***	Yes
H6: Perceived ease of use reveals positive effects on attitude	0.579	9.104 ***	82.876 ***	Yes
H7: Perceived usefulness appears positive effects on attitude	0.600	9.611 ***	92.363 ***	Yes
H8: Attitude presents positive effects on intention to use	0.647	10.866 ***	118.068 ***	Yes
H9: Technology anxiety shows moderating effects on attitude and actual behavioral intention to use	−0.191	−3.251 ***	53.687 ***	Yes

***: *p* < 0.001.

**Table 6:** Regression analysis of technology anxiety towards attitude and intention to use.

Variable	Model 1		Model 2		Model 3	
	*β*	*t*	*β*	*t*	*β*	*t*
attitude	0.647	10.886 ***	0.612	10.571 ***	0.550	9.227 ***
technology anxiety			0.221	3.815 ***	0.245	4.316 ***
attitude × technology anxiety					−0.191	−3.251 ***
F	118.068 ***		71.190 ***		53.687 ***	
R^2^	0.415		0.460		0.489	
△ R^2^	0.419		0.480		0.032	

***: *p* < 0.001.

## Appendix A

**Table A1:** The list of questionnaire items.

Dimension		Questionnaire Items
technology readiness	optimism	I know well of my health state with smart bracelets.
		2. I am interested to use smart bracelets.
		3. I believe that smart bracelets would follow my instruction.
	innovativeness	4. I would like to buy smart bracelets.
		5. I can use smart bracelets without others’ help.
		6. I am usually the first one among my friends using technological products.
	discomfort	7. Many functions of smart bracelets are not handy.
		8. The professional terms in smart bracelets are hard to comprehend.
		9. It would need a lot of time for learning smart bracelets.
	insecurity	10. I worry about smart bracelets leaking my personal information.
		11. I worry about others seeing my health information with smart bracelets.
		12. The data displayed on smart bracelets are correct.
technology interactivity	feedback	13. Smart bracelets would quickly process input data.
		14. The data display on smart bracelets is fast.
		15. I can quickly search the required information through smart bracelets.
	control	16. I have large autonomy to use smart bracelets.
		17. I am aware of the functions of smart bracelets.
		18. I can easily select the functions of smart bracelets.
		19. I can freely select the desired information from smart bracelets.
	entertainment	20. It is attractive to use smart bracelets.
		21. It is interesting to use smart bracelets.
		22. It is pleasant to use smart bracelets.
	connection	23. I would introduce the advantages of smart bracelets to others.
		24. I would recommend smart bracelets when people ask me.
		25. I would encourage relatives and friends to use smart bracelets.
perceived usefulness		26. The use of smart bracelets could increase the health control efficiency.
		27. The use of smart bracelets could effectively improve my health.
		28. The use of smart bracelets could promote the quality of life.
perceived ease of use		29. It is easy to learn the use of smart bracelets
		30. Smart bracelets provide clear instructions.
		31. I would not spend too much time using smart bracelets.
attitude		32. Smart bracelets are reliable.
		33. The use of smart bracelets is a good idea.
		34. I am satisfied with the use of smart bracelets.
intention to use		35. I expect to use smart bracelets.
		36. It is possible that I would use smart bracelets in the future.
		37. I have high intention to use smart bracelets.
technology anxiety	anxiety about equipment operation	38. I am afraid of breaking smart bracelets when I have the chance to use them.
		39. I feel clumsy when others talk about smart bracelets.
		40. I worry about not understanding the use of smart bracelets.
	anxiety about information exposure	41. I worry about the leak of personal data when using smart bracelets.
		42. I worry about smart bracelets not being able to properly protect personal health information.
		43. I am insecure about transmitting data through smart bracelets.
